# Gene purging and the evolution of Neoave metabolism and longevity

**DOI:** 10.1016/j.jbc.2023.105409

**Published:** 2023-10-31

**Authors:** Deanna Ng, Judy Pawling, James W. Dennis

**Affiliations:** 1 Lunenfeld-Tanenbaum Research Institute, Mount Sinai Hospital, Toronto, Ontario, Canada; 2 Department of Molecular Genetics, University of Toronto, Toronto, Ontario, Canada; 3 Department of Laboratory Medicine and Pathobiology, University of Toronto, Toronto Ontario, Canada

**Keywords:** essential amino acids, metabolism, longevity, Neoaves, evolution, energetics

## Abstract

Maintenance of the proteasome requires oxidative phosphorylation (ATP) and mitigation of oxidative damage, in an increasingly dysfunctional relationship with aging. SLC3A2 plays a role on both sides of this dichotomy as an adaptor to SLC7A5, a transporter of branched-chain amino acids (BCAA: Leu, Ile, Val), and to SLC7A11, a cystine importer supplying cysteine to the synthesis of the antioxidant glutathione. Endurance in mammalian muscle depends in part on oxidation of BCAA; however, elevated serum levels are associated with insulin resistance and shortened lifespans. Intriguingly, the evolution of modern birds (Neoaves) has entailed the purging of genes including *SLC3A2*, *SLC7A5, -7, -8, -10*, and *SLC1A4, -5*, largely removing BCAA exchangers and their interacting Na^+^/Gln symporters in pursuit of improved energetics. Additional gene purging included mitochondrial BCAA aminotransferase (*BCAT2*), pointing to reduced oxidation of BCAA and increased hepatic conversion to triglycerides and glucose. Fat deposits are anhydrous and highly reduced, maximizing the fuel/weight ratio for prolonged flight, but fat accumulation in muscle cells of aging humans contributes to inflammation and senescence. Duplications of the bidirectional α-ketoacid transporters *SLC16A3*, *SLC16A7*, the cystine transporters *SLC7A9*, *SLC7A11*, and N-glycan branching enzymes *MGAT4B*, *MGAT4C* in Neoaves suggests a shift to the transport of deaminated essential amino acid, and stronger mitigation of oxidative stress supported by the galectin lattice. We suggest that Alfred Lotka’s theory of natural selection as a maximum power organizer (PNAS 8:151,1922) made an unusually large contribution to Neoave evolution. Further molecular analysis of Neoaves may reveal novel rewiring with applications for human health and longevity.

Lifespan extension has been observed with calorie restriction, genetic variants and drugs that reduce mTOR signaling, and dietary restriction of branched-chain amino acids (BCAA: Leu, Ile, Val) (1, 2). Elevated levels of serum BCAA are associated with metabolic stress and insulin resistance (3, 4, 5). Mendelian randomization studies indicate that insulin resistance is causal in the elevation of serum BCAA and in turn, the risk of type 2 diabetes (6). BCAA contributes to oxidative phosphorylation, lipogenesis, the sterol pathway, glucogenesis, ketogenesis, thermogenesis, and mTOR signaling; a complexity that is not fully understood (7, 8, 9). Enzymes in the essential amino acid (EAA) synthesis pathways have been lost with metazoan evolution, perhaps minimizing stresses associated with their catabolism (10). For example, short-chain fatty acids and intermediates of BCAA catabolism may compete for carnitine and interfere with fatty acid metabolism (11, 12). BCAA catabolism generates labile acyl-CoA intermediates that decay and react with Lys residues in mitochondrial enzymes and nuclear proteins (13, 14). These auto-catalytic modifications depend on local concentrations of acyl-CoAs, and like ROS, may have both regulatory as well as adverse effects (15). High levels of other EAA can also be problematic. For example, His catabolism requires 10-formyl-tetrahydrofolate and competes with purine biosynthesis, lipogenesis, and redox control for this substrate (16, 17). Trp is catabolized by the kynurenine pathways in the synthesis of NAD^+^, serotonin, and melatonin. Kynureninase (*KYNU*) expression and the kynurenine/Trp ratio increases with age in humans, and experiments in *Caenorhabditis elegans* indicate a causal role in aging (18, 19).

SLC3A2 (4F2hc, CD98) is a type 2 transmembrane glycoprotein that forms disulfide-linked heterodimers (indicated by ∗) with the SLC7A family of EAA/Gln exchangers (SLC7A5, -6, -7, -8, -10) and the cystine/Glu exchanger (SLC7A11) that supplies cysteine to glutathione (GSH) synthesis (20, 21, 22). The evolved position of N-glycosylation sites on human SLC3A2 plays a critical role in heterodimer interactions with galectin-3 and other transporters at the cell surface (Zhang *et al*. 2023, companion paper) (23). In replete conditions, N-glycan-dependent interactions between SLC3A2∗SLC7A5 and AA/Na^+^ symporters balance EAA and Gln levels, in a continuous cycle that consumes ATP to maintain the Na^+^/K^+^ gradient. Upon oxidative stress, the interactions shift to SLC3A2∗SLC7A11, CD44, and AA/Na^+^ symporters, which supply cystine and Glu flux to GSH synthesis (24).

Herein we report that *SLC3A2*, *SLC7A* EAA/Gln exchangers, and interacting Na^+^/AA symporter genes are absent in modern birds (Neoaves), suggesting a rewiring of EAA and nitrogen metabolism. While the cystine/Glu exchangers *SLC7A11* and *SLC7A9* genes are present in duplicate, suggesting an enhanced capacity to suppress oxidative stress. In parallel, “Kelch Like ECH Associated Protein 1” (*KEAP1*) is absent, an E3 ubiquitin ligase that targets the transcription factor NRF2, a key promoter of antioxidant genes (25). Loss of *KEAP1* enhances NRF2-driven expression of SLC7A11 as well as key enzymes in GSH synthesis (GCLC, GCLM) and the thiol redoxins (TXNRD1, PRDX1). Many Neoave species display extended lifespans relative to body size (26). Lifespan extension can be achieved in mice by single gene deletions that reduce insulin/mTOR signaling (27), or deletions that reduce ROS (*e.g. KEAP1, p66SHC*) (28, 29, 30). Canine breeds selected for small size also show extended lifespans and mutations in the insulin pathway (31, 32, 33, 34). However, Neoave genomes may reveal higher-dimensional gene interactions beyond what can be achieved with inbred strains of mice and dogs. The evolution of birds may be viewed as a gain-of-function, gene drop-out experiment driven by adaptation to a volant lifestyle, which has resulted in a remarkable radiation of species occupying ecosystems worldwide and many with extended longevity. Lotka viewed natural selection through the lens of open-system thermodynamics: - organisms that best capture and maximize the utility of available energy hold an evolutionary advantage (35). In this preliminary report, we focus on how the selective purging of genes may have contributed to fitness and longevity in Neoaves.

## Results and dissusion

### Lightening the load by purging genes

Aves emerged from bipedal dinosaurs ∼165 to 150 million years ago (MYA), survived the Cretaceous–Paleogene extinction event 66 MYA, and then diversified into the ∼10,000 Neoaves species we observed today (36). Phylogenetic analysis of Neoaves suggests a massive convergence of protein-coding sequences, and incomplete lineage sorting during rapid radiation between 60 to 50 MYA (37). The benefits of becoming endothermic, smaller, and adapted for flapping-wing flight allowed for greater foraging opportunities, predator avoidance, and tolerance to a great range of environments (26, 38, 39). The power required to fly long distances is largely a multiple of basal metabolic rates (BMR), and smaller birds with proportionately more fat reserves can fly longer distances than large birds^1^. Indeed, genes involved in energy metabolism show strong evidence of positive selection, suggesting early adaptative mutations required for flight (40). Body mass correlates with BMR and longevity, although shifts and variations across vertebrate phylogeny remain unexplained (41). Many Neoaves are outliers, showing greater longevity and higher BMR than expected relative to body size (25, 26).

Genome expansion occurred with the emergence of eukaryotic cells and again in metazoans, perhaps limited at some point by two factors; bioenergetics and systemic complexity (42). Complex self-organizing systems from species to empires are driven by node expansion and connectivity, ultimately becoming less resilient to damage and the disorder associated with their aging (43, 44, 45). Assuming most species are near this type of boundary condition, natural selection in a novel environment may meet this challenge by purging genes, thereby increasing fitness by streamlining the interdependencies and reducing the cost of maintenance. Indeed, selective pressures leading to a volant lifestyle drove a surprising ∼25% reduction in Neoave genome size and ∼10% in bats (46, 47, 48). This has reduced apparent redundancies by purging gene paralogues, resulting in compartmentalization that may improve bioenergetics, as discussed below. Gene purging has not disadvantaged Neoave, as many species display larger brains relative to body size, sharper senses and memory, and complex vocalization, and some species use tools and pass knowledge between generations (36).

**Figure 1:**
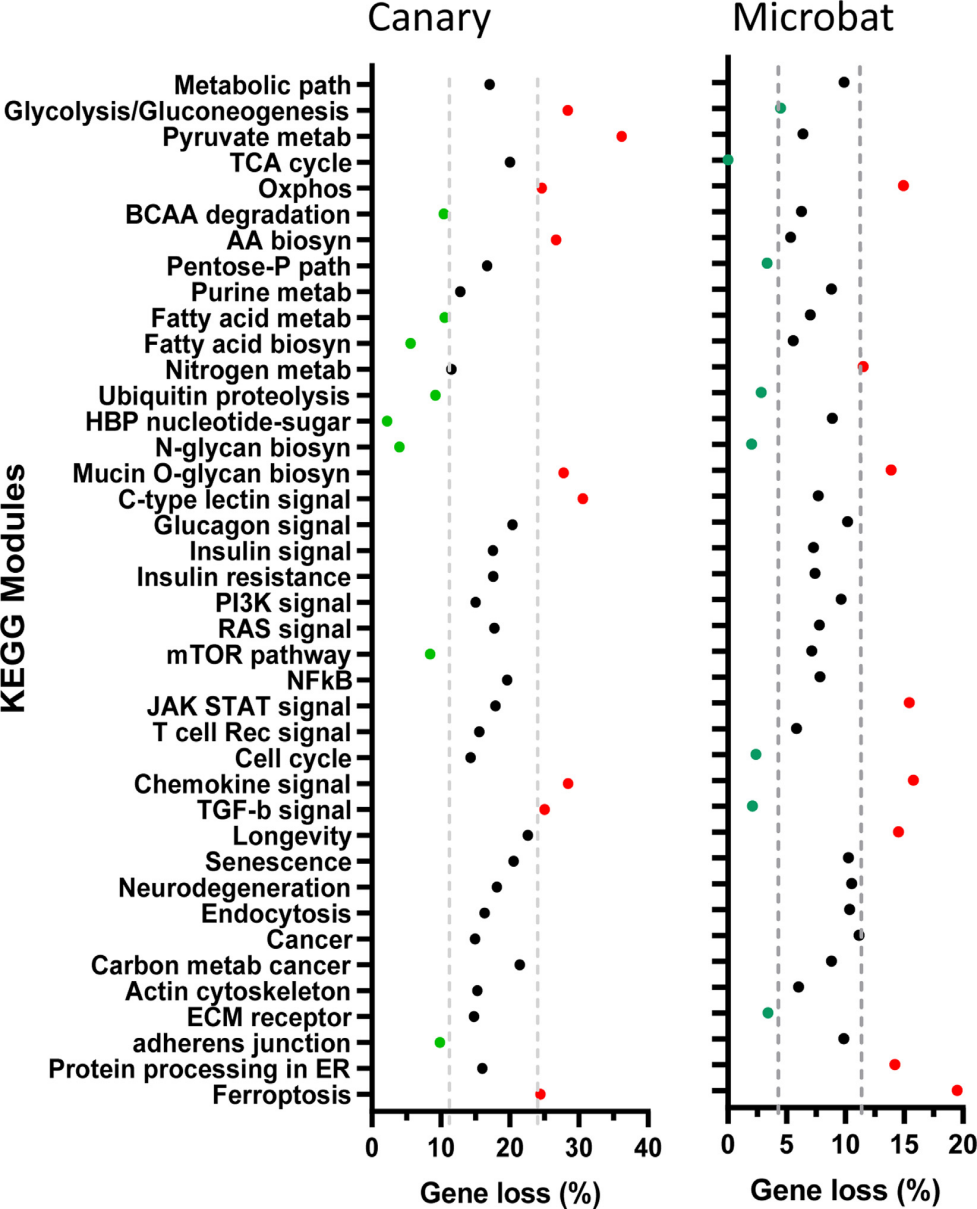
**Fraction of genes missing from 45 KEGG modules.** gProfiler was used to search Serinus canaria (canary) and myotis lucifugus (microbat) genomes to determine the absent genes in each KEGG module, expressed as a percentage. The 45 modules have a total of 3730 nonredundant human genes (Table S1). *Dashed lines* are 3 SD from the mean of the 45 modules.

The canary (*Serinus canaria*, a Neoave) and chicken (*Gallus gallus*, a progenitor lineage), have an estimated 15,281 and 17,478 protein-encoding genes, respectively. Human and mouse (*Mus musculus*), separated by a similar ∼80 MYA, have ∼22,389 and ∼23,317, respectively, with > 98% of the genes functionally annotated by homology (49). Gene loss (mean ± SD) in *Serinus canaria* and Microbat (*Myotis lucifugus*) for 45 KEGG modules of interest was 17.4% ±3.67 and 8.4% ±3.4, respectively (Fig. 1 and Table S1). Bats are the only mammal to have evolved flapping-wing flight, which also led to smaller genomes and extended life spans (26, 47). Microbat has *SLC3A2* and associated transporter genes but has lost clinically interesting genes in common with those of Neoaves including *BCAT2*, *GCKR*, *FOXO1*, *FOXO6*, *GSK3A*, *AKT1*, and *TP73* (*AKT2* and *TP53* are lost in Neoaves). However, our focus here is on Neoaves where greater losses are observed (Fig. 1); exemplified by pyruvate metabolism (KEGG:00,620); glycolysis/gluconeogenesis (00,010); and oxidative phosphorylation (00,190). KEGG modules with more genes retained include the hexosamine biosynthesis pathway (00,520); N-glycan biosynthesis (00,510); fatty acid biosynthesis (01,212); mTOR pathway (04,150); ubiquitin proteolysis (04,120). Senescent cells over-express cyclin-dependent kinase inhibitors p21 and p16, which are associated with chronic tissue inflammation and dysfunction. Targeted elimination of these p16^+^ cells in mice increases lifespan and prolongs physical vitality (50). Intriguingly, Neoaves have purged *CDKN1A* (P21) and *CDKN2A* (P16) as well as two associated kinases *CDK2* and *CDK4* that also play a role in metabolism, cell cycle and gene expression.

### Energy expenditure on maintenance

Three activities make major contributions to energy expenditure on maintenance in non-dividing cells: 10 to 25% for protein turnover, 10 to 30% by Na^+^/K^+^ ATPase exchange and ∼20% by mitochondrial proton leakage (51). Presumably, gene purging reduces the energy cost of maintaining a larger than needed genome and proteome. Indeed, longevity is negatively correlated with rates of protein turnover measured in confluent cultures of primary fibroblasts from diverse mammalian species. Swovick at al (52). conclude that fast protein turnover is sufficient in shorter-lived organisms, but the consumption of ATP and generation of ROS over time would disadvantage longer-lived species. Thus, the beneficial effects of protein turnover on proteostasis are balanced against the energy costs of protein re-synthesis and the associated oxidative damage that accumulates with aging. As such, Neoave longevity may arise from a reduced cost of maintenance; fewer genes and slower protein turnover made possible by enhanced suppression of ROS (25), and an evolved resistance to protein glycation (53, 54).

**Figure 2:**
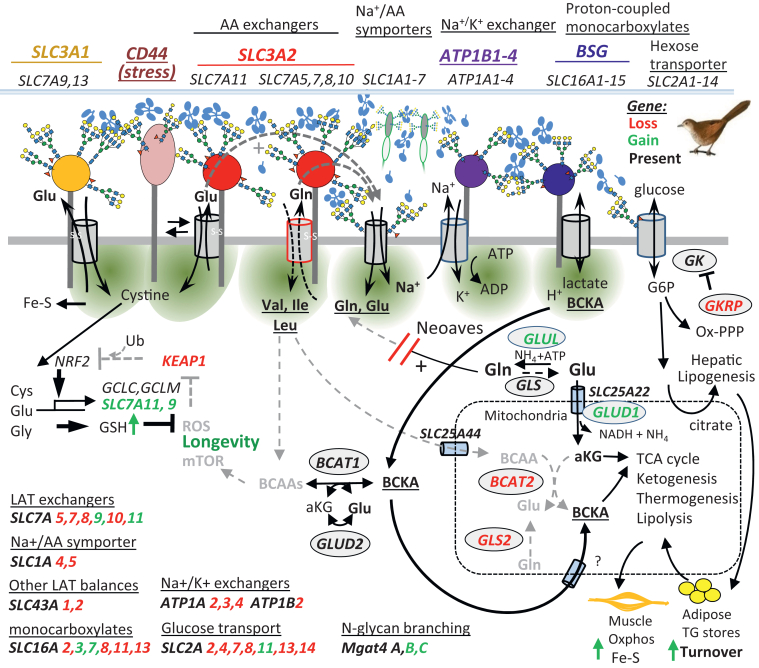
**Transporter network in mammals; gene loss and gain in Neoaves.** Uptake of EAA and cystine by SLC3A2∗SLC7A exchangers driven by Gln and Glu (*dashed arrows* above the membrane) is enhanced by N-glycans-dependent clustering by Galectin-3 (*blue pentamers*). Galectins *LGALS1, -2, -3* and *-8* are conserved in Neoaves. EAA import promotes mTORC1 activation. Purging *SLC3A2*, Gln-driven *SLC7A* exchangers, and a *SLC1A4,-5* Na+/AA symporters in Neoaves is likely to reduce energy consumption by Na+/K+ ATPase exchanger. Loss of *BCAT2*, *GLS2*, and duplications of *SLC16A1* and of *SLC16A3* bidirectional proton-coupled monocarboxylate transporters may promote branched-chain ketoacids (BCKA) exchange and flux into triglyceride and adipose stores. The remaining adaptors SLC3A1, BSG, and ATP1B1,3,4 can also be cross-linked by galectins in the regulation of an alternate transporter network in Neoaves. N-glycosylation pathways are enhanced by duplications of *MGAT4B* and *MGAT4C* supporting a higher affinity galectin lattice. With duplications of *SLC7A9, SLC7A11* and loss of *KEAP*, the supply of cystine to GSH and Fe-S complex is supercharged.

Saving energy is possible by reducing the activity of Na^+^-driven symporters and their burden on Na^+^/K^+^ ATPase exchangers. The export of Gln with the import of EAA by SLC3A2∗SLC7A exchangers (55, 56) requires the recovery of Gln by AA/Na^+^ symporter and maintenance of the Na^+^/K^+^ gradient by ATPase exchangers (Fig. 2). Selection against this apparent burden on energetics in Neoaves is supported by the loss of *SLC3A2*, *SLC7A7*, *SLC7A8*, *SLC7A10*, and N-terminal truncations of *SLC7A5* that remove four or five TM domains, predicted to disrupt the hydrophobic core of the protein fold (Fig. S1, *A*–*C*). Two other sodium-independent large amino acid transporters, *SLC43A1* and *SLC43A2* are also absent, leaving *SLC6A15* encoding a known transporter of BCAA found in the mammalian brain, and *SLC7A6*, an antiporter exporting Arg and Lys in exchange for Leu, Ile and Gln also expressed in brain and cancers. SLC3A2∗SLC7A5 is functionally associated with the Glu and Gln AA/Na^+^ symporters SLC1A4 and SLC1A5 in BCAA/Gln (55), which are also absent in Neoaves. Thus, the purging of nine genes encoding the EAA/Gln exchanger – AA/Na^+^ symporter module of regulation, suggesting an alternate means of regulating EAA and nitrogen metabolism as discussed below.

If fitness is improved by conserving ATP, then losses of paralogs by >25% might also be expected in other ATPase-dependent transporter families and inward Na^+^ channels. Indeed, 38% (16/42) ATPase-dependent ABC transporters are absent or severely truncated, and the same for 64% (9/14) of the *SLC9A* family of Na^+^/H^+^ exchangers, a proton extruding system driven by the inward Na^+^ gradient. In the *SCN1A to -11A* family of voltage-dependent Na^+^ permeability channels, 82% (9/11) are absent or truncated, and only duplicated *SCN2A* and *SCN5A* genes are present. Similarly, 85% (6/7) of the purinoreceptors (*P2RX1-7*), a family of ATPase-gated cation-permeable ion channels (Ca^++^/K^+^/Na^+^) are also truncated or missing in Neoaves. Only *P2RX5* is well conserved in birds (∼93%) and upstream ATP-channels (*PANX1-3*), downstream ectonucleotides *(ENPP-1, 3*) and receptors (*P2RY-1, 2*) are present suggesting the pathway is intact.

**Figure 3:**
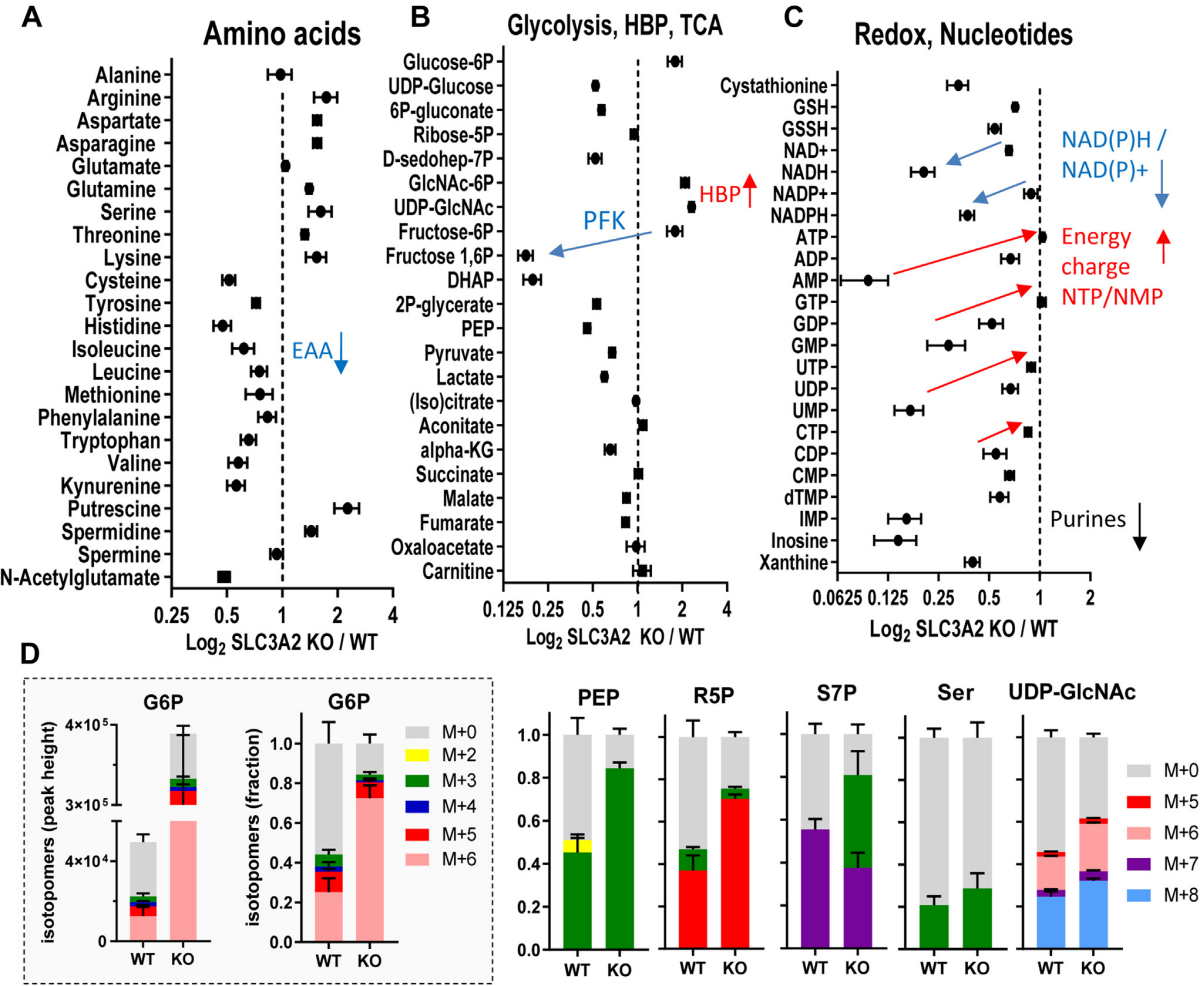
**SLC3A2 mutation in HeLa cells alters central metabolism.***A–C*, intracellular metabolites by LC-MS/MS displayed as SLC3A2 KO/WT ratios, mean ± SD (n = 6) biological replicates cultured in DMEM + 10% FBS. Phosphofructokinase (PFK). *D*, cells were cultured in [U-^13^C]-glucose for 24h to label downstream metabolites. G6P in the box is displayed as mass intensity/cell (*left*) and fractional distribution of ^13^C-label (*right*). Phosphoenolpyruvate (PEP), ribose 5-phosphate (R5P), sedoheptulose 7-phosphate (S7P), Serine (Ser), uridine diphosphate-N-acetylglucosamine (UDP-GlcNAc). The phenotype of SLC3A2 deficient HeLa cells has been characterized in more detail (Zhang *et al*. 2023, companion paper)^1^.

Proton leakage in mammalian mitochondria represents a loss to ATP production and may contribute to ROS production (51). In this regard, SLC3A2 KO HeLa cells display features that may be beneficial in Neoaves. IMP, AMP, and GMP levels were depleted in SLC3A2 KO cells; however, ATP levels were maintained, suggesting an enhanced coupling of oxidative phosphorylation (Fig. 3, *A*–*C* and Table S2). FLAG-SLC3A2 interacted with the mitochondrial ATP synthesis complex V in AP-MS experiments perhaps reducing coupling (Zhang *et al*. companion paper). Fructose-6P was 4-fold higher and fructose 1,6-bisphosphate (F1,6BP) ∼8 times lower in SLC3A2 KO cells, indicating a decrease in phosphofructokinase activity that increases glucose-6P flux to the oxidative pentose phosphate pathway (ox-PPP), and fructose-6P into the hexosamine biosynthesis pathway (Figs. 3 and S2*A*). Glucose flux to ox-PPP supplies NADPH to antioxidant and lipogenesis pathways, which is likely to be advantageous in Neoaves. Low levels of F1,6BP provide a means of activating AMPK independent of the AMP/ATP ratio (57) (Fig. S2, *B*–*D*). Activation of AMPK opposes oxidative stress by increasing glucose flux, and mitochondrial biogenesis, which is also likely to be an advantage in Neoaves (58). Other cell and animal models will be required to experimentally unravel how natural selection has shaped Neoave metabolism.

### Regulation of BCAA in Neoaves

Mitochondrial BCAA aminotransferase (*BCAT2*) is absent in all Aves, leaving only cytoplasmic *BCAT1* to balance BCAA levels. The BCATs catalyze bidirectional transamination between BCAA + α-ketoglutarate ↔ branched-chain α-keto acids + Glu (BCKA + Glu). *BCAT2* is expressed widely in human tissues, while *BCAT1* is highest in brain, kidney, and upregulated with cancer progression (59). In *BCAT2* deficient mice, plasma BCAA levels and energy expenditure are increased, adiposity decreased, and insulin sensitivity improved (60). A similar phenotype was observed in mice deficient in hepatic mitochondrial glutaminase (*GLS2*) (61), a gene that is also absent in Neoaves. Endurance is reduced in mice with *BCAT2* or *SLC7A5* mutations (62, 63), consistent with a need for BCAA oxidation in mammalian muscle. However, endurance in Neoaves may be enhanced with loss of *BCAT2*, *GLS2*, and *SLC7A* genes, by reducing BCAA uptake and oxidation in muscle, while increasing hepatic conversion of BCKA to triglycerides that can supply ketones during flight (Fig. 2). Fat is anhydrous and the highest density of stored energy, thus minimizing the weight to fuel ratio for prolonged flight. Muscle glycogen synthesis (*GYS1*) is absent, presumably replaced by higher levels of circulating glucose (46) and by mobilization of fatty acids during flight. Carnitine O-palmitoyltransferase 1a (*CPT1A*, kidney, heart, liver) is present, and *CPT1B/C* (muscle, brain) is absent, consistent with the liver as the major supplier of ketones to other tissues. Mitochondrial pyruvate carboxylase (*PC*) is absent, an enzyme required to transfer citrate-derived oxaloacetate from the cytoplasm into the mitochondria, thus oxaloacetate is more likely redirected into gluconeogenesis and PPP. Moreover, phosphoenolpyruvate carboxykinase (*PCK2*) is also absent, leaving cytoplasmic *PCK1* to mediate the reversible conversion of phosphoenolpyruvate and oxaloacetate, a key factor in flux of carbon between glucose, PPP and lipid metabolism.

**Figure 4:**
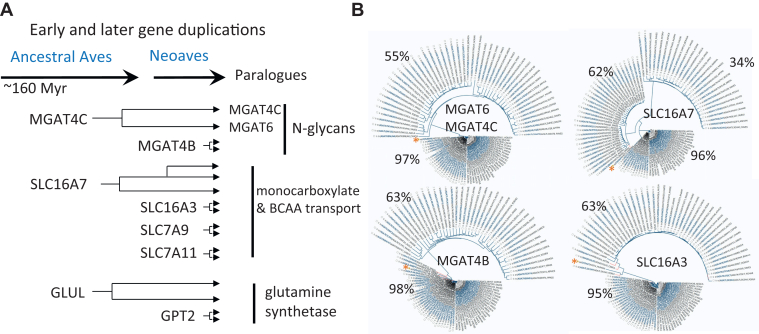
**Examples of gene duplication in Neoaves.***A*, these examples are discussed in the text, N-glycan branching, BCKA transporters and nitrogen metabolism. *B*, phylograms generated using Uniprot (Ave 8782) species listed in alphabetical ordered and taking the first 100 sequences (*i.e.* 2 or 3 paralogues per species). The approximate percent identities within paralogue clusters are indicated. The phylograms are typical examples of a conserved paralogue closer to the human gene and a rapidly evolving paralogue. ∗Human sequence. Single gene orthologues are present in Zebra fish, consistent with ancient origins of these genes. There is experiential evidence for functional divergence for one of the *MGAT4C* paralogue - *MGAT6*, Uniprot: Q9DGD1 (68, 69).

Monocarboxylates including BCKAs and ketones are substrates of the SLC16A family of bidirectional proton-coupled monocarboxylate transporters. SLC16A3 and SLC16A7 are present as two and three paralogs in Neoaves, respectively (Fig. 4). This suggests that the SLC16A family of transporters may be sufficient to balance the keto-forms of EAA and their re-amination as needed for protein synthesis and other pathways. In mammals, dietary proteins are hydrolyzed to small peptides and AA, taken up in the small intestine, and transit to the liver where both BCAT1/2 activities are silenced and BCAA are released to the circulation. However, BCAT activity in muscle is high which converts BCAA and releases BCKA to the circulation (59). The activation of BCAT1 in Neoave liver and/or small intestine might allow for the release of BCKA into the circulation and promote metabolic conversion in the liver. In tissues more widely, a lower BCAA/BCKA ratio may contribute to Neoave longevity by reducing basal mTOR activity concordant with lower protein turnover. Thus, deaminated BCAA and the other EAA (α-ketoacids) are likely to be the primary “currency” of exchange between tissues, mediated by the SLC16A transporters and its BSG adaptor, perhaps aided by the strengthened galectin lattice (Fig. 2).

The purging of *SLC3A2* and associated transporters in Neoaves predicts a shift to the exchange of BCKA which no longer requires Gln as drivers of EAA uptake, perhaps allowing greater flux of Gln to ammonia disposal. A duplication of the glutamine synthetase gene (*GLUL*) supports ATP-dependent catalysis of NH_4_ + Glu → Gln, and flux into the purine pathway to uric acid. Bird physiology conserves water by shifting ammonia disposal to this less soluble end-product, excreted as guano; ∼10% protein and ∼80% uric acid in colloidal suspension. In addition, uric acid acts as an antioxidant that activates the NRF2 pathways (64), working in parallel with the loss of *KEAP1* (25). Histidine also has antioxidant activity (65), and loss of function mutations in human histidine ammonia lyase (HAL), the first enzyme in the catabolic pathway is associated with a reduced hazard of coronary heart disease (17). HAL is absent in Neoaves, presumably reducing both the production of ammonia and the consumption of 10-formyl-tetrahydrofolate by the purine pathway.

### ROS suppression and N-glycans

In the control of ROS, gene duplication of the cystine transporter SLC7A11 and a conserved association with CD44 may compensate for the loss of SLC3A2 (24) (Fig. 2). Moreover, the *SLC7A9* gene is present in duplicate, a transporter that heterodimerizes with SLC3A1 (rBAT) and re-absorbs cystine in kidney tubules (66, 67). Unlike its paralogue, SLC3A1 is conserved in Neoaves and has six N-glycan sites but lacks the ubiquitination sites at the N-terminus. Clustering SLC3A1∗SLC7A9 with AA/Na^+^ symporters and ATP1B- associated transporters by galectins is expected to be higher affinity due to *MGAT4B* and *MGAT4C* gene duplications (Fig. 4). Notably, one of the MGAT4C paralogues (MGAT6, Uniprot: Q9DGD1) evolved a novel specificity that adds N-glycans branch not found in mammals (68, 69). Ectopic expression of MGAT6 in HeLa cells generated penta-antennary N-glycans and enhanced central metabolism. The N-glycan branching pathway promoted metabolite [^15^N]-labeled Gln import, higher EAA levels, and cell growth in low glucose/Gln medium (70). Indeed, hexosamine biosynthesis and N-glycosylation pathways are conserved in Neoaves. Thus, a stronger galectin lattice is expected to enhance cell surface retention of SLC3A1∗SLC7A9 and provide Cys to GSH, iron-sulfur clusters (Fe-S), and Coenzyme A synthesis, linking stress mitigation and TCA cycle, respectively (71). In mice, N-glycan branching promotes growth factor receptor signaling, maintenance of stem cells, soft tissue mass (muscle), skeletal integrity, and lifespan (72, 73).

**Figure 5:**
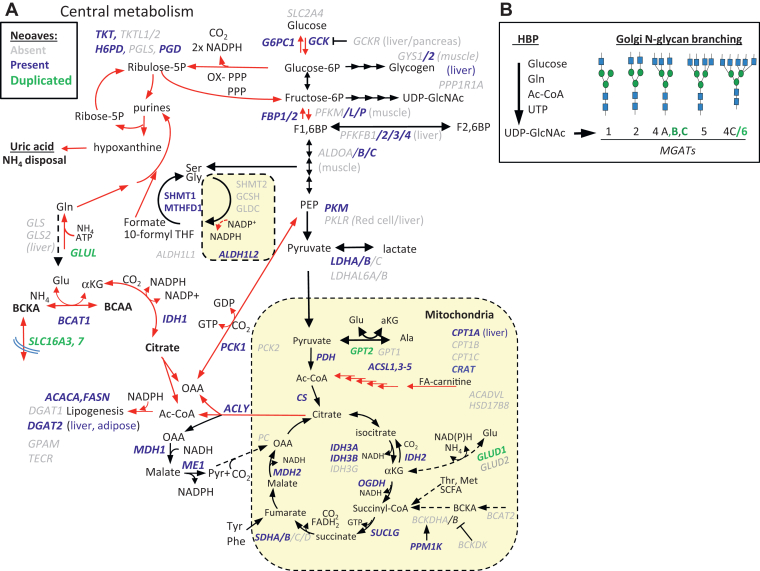
**Altered Metabolism in Neoaves.***A*, genes present (*blue*), absent (*grey*) or duplicated (*green*) in Neoaves. *Red arrows* are likely enhanced routes of flux in liver, due in part to losses of GCKR, PC, PCK2, BCAT2 and GLS2. Other proteins associated with BCAA dehydrogenase complex are present; ACAD8, ACADSB, HIBADH, HIBCH, IVD and DLD, while HSD17B10 is two-thirds truncated, a 3-hydroxy-2-methylbutyryl-CoA dehydrogenase in branched-chain amino acid catabolic pathway. See Fig. S3 for gene-enzyme list. *B*, the Hexosamine biosynthesis pathway (HBP) competes with central metabolism for substrates in the synthesis of UDP-GlcNAc which is rate-limiting for the Golgi N-glycan branching pathway (90).

As described above, streamlining paralogues including *GYS1*, *CPT1B/C*, *PCK2*, *ADHL1L1/2*, and SHMT2 is consistent with this altered pattern of glucose flux in tissues and between glycolysis, ox-PPP, and the one-carbon and TCA cycles (Figs. 5 and S3). The insulin-inducible glucose transporter (*SLC2A4*) is absent in Neoaves and circulating glucose is 2- to 3-fold higher than in mammals (46). The Advanced Glycation End product-specific Receptor (*AGER*) is absent in Neoaves, which internalizes AGE-damaged extracellular matrix proteins for degradation in mammals (74). Protein glycation occurs at Lys residues, and a multispecies comparison of serum albumin revealed lower numbers of exposed Lys residues in birds than in mammalian sequences, suggesting an evolved resistance to high serum glucose (53, 54), perhaps increasing the half-life of proteins as discussed above. The absence of glucokinase regulatory protein (*GCKR*) is predicted to weaken the stringency of switching from gluconeogenesis to glycolysis, thus increasing fasting glucose levels, and promoting lipogenesis upon feeding. Common loss-of-function variants in *GCKR* have been linked with elevated hepatic cytosolic NADH/NAD^+^ ratio (*i.e.* reductive stress) and ∼130 metabolic traits, including elevated serum glucose, BCAA, α-hydroxybutyrate, triglycerides, and insulin resistance (75).

**Figure 6:**
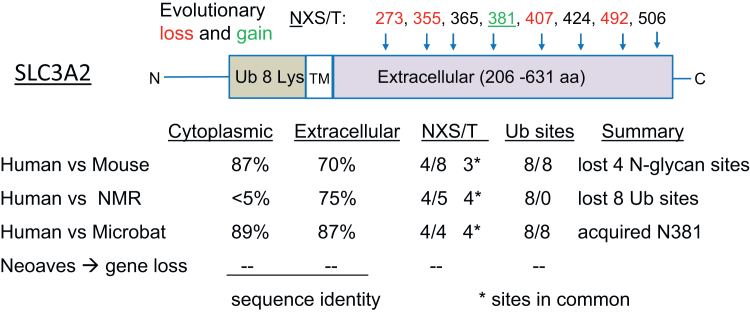
**Divergent evolution of SLC3A2.** Sequence identity in the extracellular and cytoplasmic domains of SLC3A2 from humans, mice, naked mole rats (NMR), and Neoaves reveals four distinct patterns.

Stringent control of ROS and minimizing the energy cost of proteome maintenance appear to be key features but longevity can emerge in different ways. Neoaves and Naked mole rats both display remarkable longevity relative to body size but differ in lifestyle and the evolutionary path to this status. Naked mole rats live underground and are adapted to low oxygen levels and a slower metabolic rate, without significant gene purging (49). They are resistant to cancer, show negligible senescence, and maintain healthy vascular function longer into their lifespan than other rodents (76, 77). Contributing factors include near error-free protein synthesis (78), increased expression of CDK inhibitors, enhanced mitigation of oxidative stress (79), and extremely high-molecular-mass hyaluronan (80) recently shown to extend life- and health-span in mice (81). The extracellular domain of SLC3A2 has five N-glycans including the primates-derived site at N381, while the cytoplasmic sequence is divergent and lacks the Lys residues that target SLC3A2 for proteolysis (Fig. 6). This suggests increased stability at the cell surface, and points to the functional importance of the extracellular domain (82, 83), which in mice, is required by metabolism in early post-implantation embryonic development (84, 85). With apparent serendipity, SLC3A2 in hominids acts as a receptor for an envelope glycoprotein encoded by the endogenous retrovirus HML-2, which stimulates mTOR activity required in brain development (86). Overexpression of HML-2 is associated with tumorigenesis and neurodegeneration, similar in this regard to overexpressing of SLC3A2 in cancers more widely (87, 88). As an opportune interaction in embryogenesis, HML-2-SLC3A2-mTOR adds risk to longevity (45).

The selection pressures leading to flight in Neoaves and bats have modified body plans, improved bioenergetics, streamlined genomes, and presumably purged burdensome genes in the process. Well-known vertebrate genes linking cell cycle, signaling, metabolism, and cellular senescence are absent in Neoaves, including *TP53*, *CDK2*, *CDK4*, *CDKN1A* (P21), *CDKN2A* (P16, P14), *AKT2*, *HRAS*, *RPS6KB2*, *FOXO1*/*6* and *GSK3A*. Most of these genes have paralogues with overlapping and unique activities, yet ultimately one or more has been purged in Neoave which may contribute to vitality into the latter years of lifespan. Thus, comparative analysis of Avian genomes and proteomes coupled with machine learning may reveal the rewiring of molecular networks with novel applications to human health.

## Experimental procedures

### Evolutionary purging and duplication of genes in Neoaves

Neoaves represent ∼95% of all avian species and the remainder are extant Ave species, the Palaeognathae [ID:8783] (ostriches and allies), and Galloanseres [ID:1,549,675] (ducks and chickens). Mammalian genes of interest were searched across ∼250 Ave species using UniProt Taxonomy - Aves [ID:8782]. The largest subgroup of ∼150 species in the UniProt Taxonomy are the Passeriformes [ID:9126] (songbirds). Full-length SLC3A2 sequences (∼500AA) are present in 2 species (kakapo and small tree-finch), truncated (52–138AA) in 41 and absent in the remainder Neoave species. Similarly, the KEAP1 gene is full length (∼600AA) in 6 species (chicken, kakapo, wild turkey, new Caledonian crow, mallard, kiwi; truncated in 28 and absent in the remaining Neoave species (25). SLC7A5 (>500Aa) is present in 22 species of Galloanseres, Palaeognathae, and a few Passeriformes, and truncated in the remaining species to ∼332AA or ∼286AA, which removed the first 4 or 5 transmembrane domains, respectively. Other genes of interest were examined in the same manner and classified as absence in Neoaves if they were not found in any Aves, or present largely in extant Aves (*e.g.* Galloanserae, Paleognathae) but severely truncated or absent in the majority (>70%) of Neoaves. g:Profiler was used to compare GO and KEGG modules in humans, serinus canaria (canary), and myotis lucifugus (microbat). Further analysis will be required to deconstruct the combinations of genes present and lost at the species level. With the large numbers of purged and retained genes, tools like gProfiler are not sufficient to extract functionality and statistical relationships.

### HeLa cells and metabolite profiling by LC-MS/MS

HeLa Flp-In T-REx cells were a kind gift from Dr Stephen Taylor (University of Manchester). HeLa Flp-In T-REx cell lines were cultured at 37 °C and 5% CO_2_ in a humidified atmosphere in high glucose DMEM (Wisent, St Bruno, Quebec) supplemented with 10% fetal bovine serum (FBS), 2 mM Gln, penicillin (50 I.U./ml)/streptomycin (50 ug/ml). The Flp-In T-REx HeLa cells (designated as WT cells below) were targeted for SLC3A2 deletion by CRISPR-Cas9 (Zhang *et al*. companion manuscript)^1^.

HeLa cells, wild type and SLC3A2 deficient were seeded in 6-well plates with 6 technical replicates per experimental condition and cultured for 24 h, followed by two quick washes of the wells with warm PBS (∼37 °C), then the plates were flash frozen in liquid nitrogen (89). The metabolites were immediately extracted by adding 1 ml of extraction solution (40% acetonitrile, 40% methanol, and 20% water) and then the cells were scraped and collected in 1.5 ml vials. Metabolite extraction and analysis by LC-MS/MS is described in detail in the companion manuscript (Zheng *et. al*. JBC 2023)(23).

## Data availability

Data was sourced from Gene bank and Uniprot as described in methods (Table S1). Metabolite data is found in Table S2. There is no other supporting data or information.

## Supporting information

This article contains supporting information:

Figure S1 SLC7A5 truncation in Neoaves

Figure S2 Metabolism.

Figure S3 Gene – enzyme list.

Table S1 Genes absent from Neoaves.

Table S2 Metabolism in SLC3A2 WT and KO HeLa cells.

## Conflict of interest

The authors declare that they have no conflicts of interest with the contents of this article.

## Footnotes

1 Ecology Center, https://www.ecologycenter.us/breeding-areas/body-weight-speed-and-flight-mode.html.
