# Supplementary material for: Gene purging and the evolution of Neoave metabolism and longevity
Source: J Biol Chem. 2023 Oct 31;299(12):105409. doi: 10.1016/j.jbc.2023.105409 (PMC10722388; doi:10.1016/j.jbc.2023.105409)
Supplement: Supporting data 1 [file mmc1.pdf]

Supporting Information

Gene purging and the evolution of Neoave metabolism and longevity.

Deanna Wan Jie Ng, Judy Pawling, , and James W. Dennis

Figure S1 SLC7A5 is truncated in Neoaves

Figure S2 SLC3A2 deletion in HeLa cells disrupts central metabolism

Figure S3 Gene – enzyme list

Table S1 Genes absent from Neoaves.

Table S2 Metabolites in SLC3A2 KO and WT HeLa cells

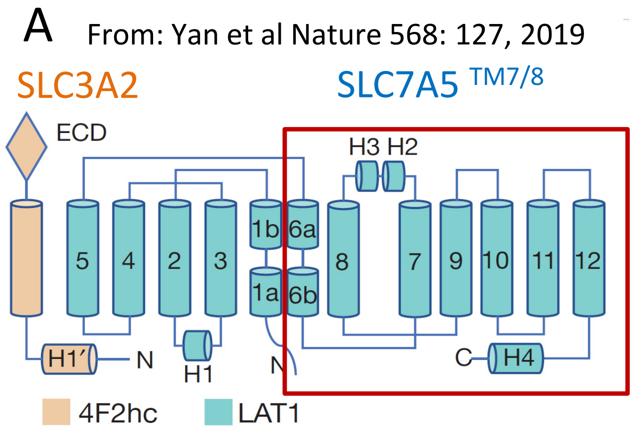

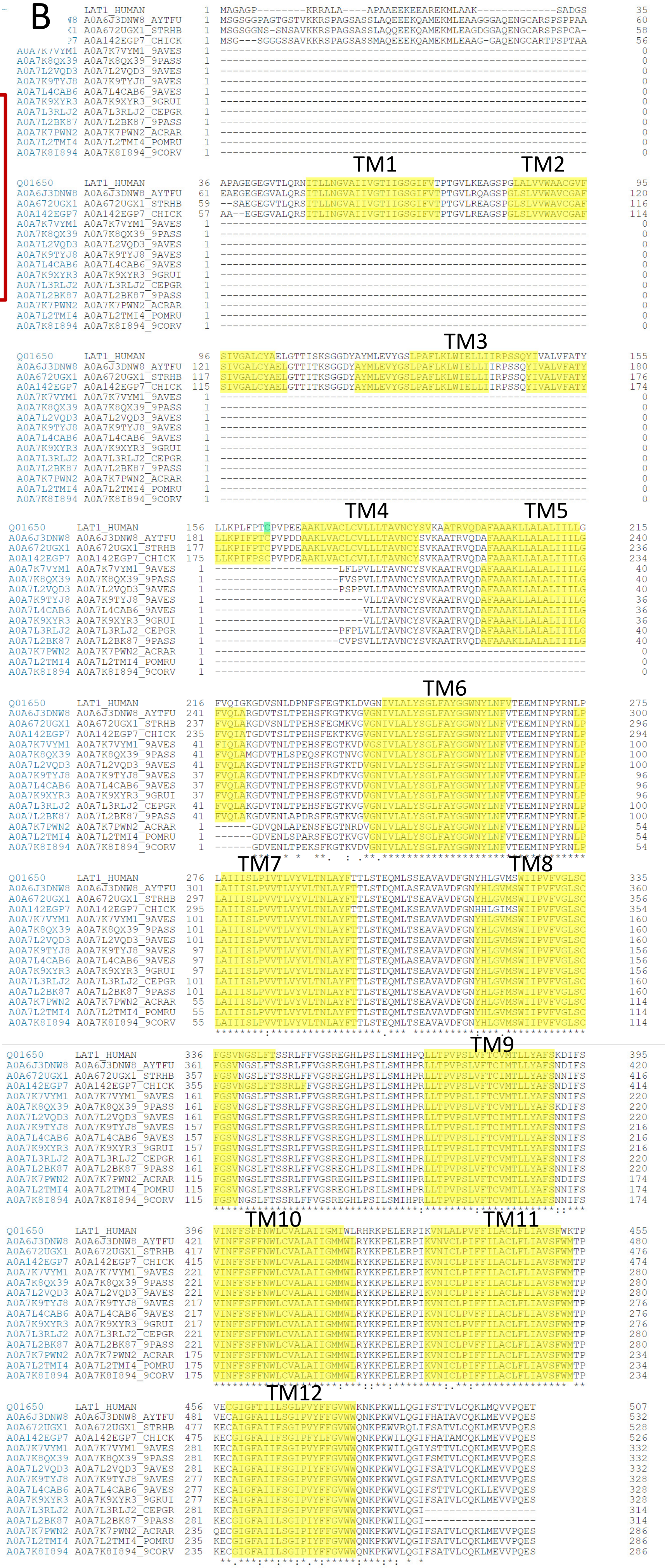

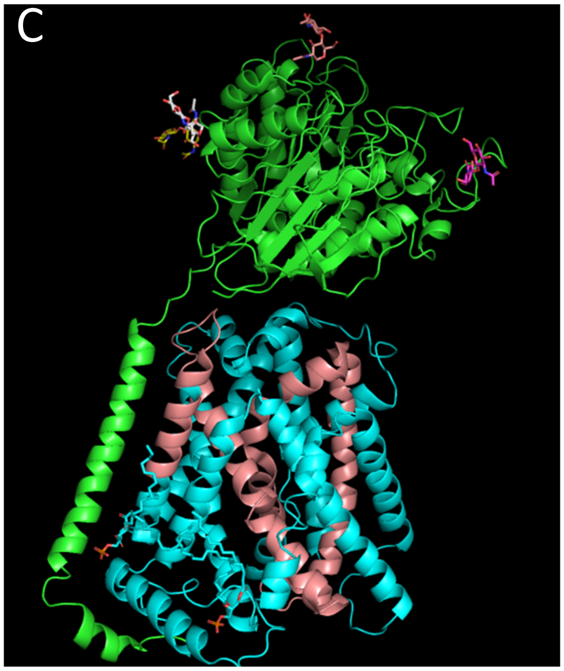

Figure S1: SLC7A5 is truncated in Neoaves. (A) SLC3A2 (green) and SLC7A5 (TM domains (blue and red) (from Yan R. et al. *Nature* 568:127, 2019) **(B)** Alignment of human SLC7A5 with bird sequences reveals the 12 TM, in 42 ancestral Aves species and truncation at the N-terminus in all Neoave species, leaving 8 or 7 TM domains; (blue in panel **(C)**. TMs 1-5 (red) appear to be critical to SLC7A5 fold, and loss is expected to disrupted the structure and eliminated activity. An alternate fold with activity is of course possible. Notably, similar truncations are rare, but observed in Protobothrops mucrosquamatus (Brown spotted pit viper). Anole Carolinensis (Green Anolis), Neotoma lepida (Desert wood rat). Castor Canadensis (an extant beaver species),and Larimichthys crocea (Large yellow croaker).

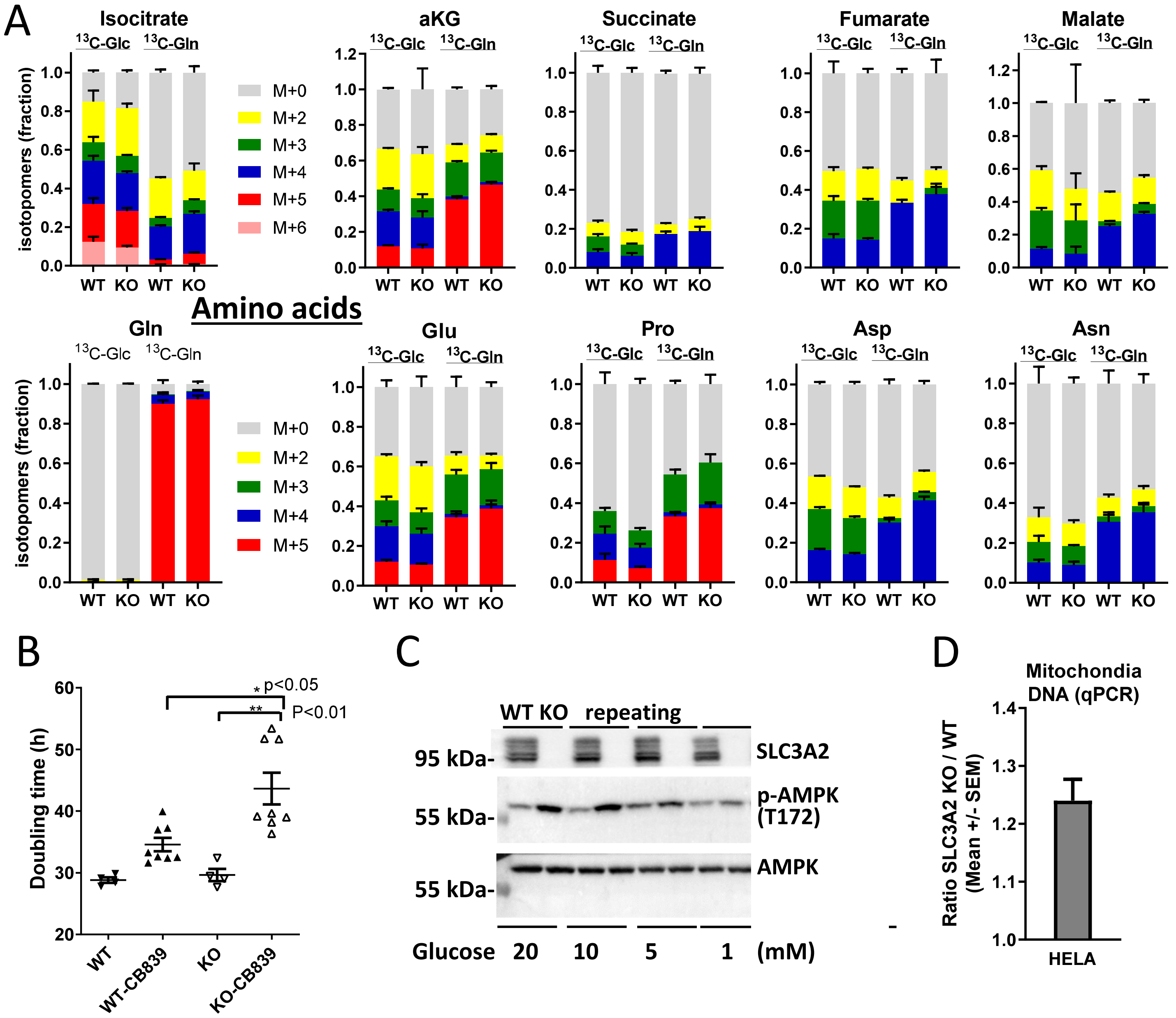

Figure S2: SLC3A2 deletion in HeLa cells disrupts central metabolism (A) SLC3A2 WT and KO HeLa cells were cultured in medium where glucose and glutamine were replaced with [U-^{13}C]-glucose or [U-^{13}C]-glutamine for 16h and the ^{13}C-labeled fractions were quantified by LC-MS/MS. The results indicate more [U-^{13}C]-glutamine flux in SLC3A2 KO cells into the TCA cycle, while [U-^{13}C]-glucose flux increased glycolysis intermediates in SLC3A2 KO cells. [U-^{13}C]-glutamine flux to Asp (M4) was higher in SLC3A2 KO cells. *The Asp M3/M4 ratios from [U-^{13}C]-glutamine labeling of Asp in KO 0.096 and WT 0.075, fumarate in KO 0.1 and WT 0, malate in KO 0.17 and WT 0.15, indicating a greater contribution of [U-^{13}C]-glutamine to reductive conversion of aKG to citrate, and oxaloacetate transamination to Asp. **(B)** Doubling time in DMEM +10% FCS without and with 0.2 μM CB839, a glutaminase inhibitor. **(C)** AMPK activation in SLC3A2 KO and WT cells cultured in DMEM 1 mM Gln +10% dialyzed FCS with 1 to 20 mM glucose for 48 h. **(D)** Relative mitochondrial content by qPCR.

Figure S3: Gene – enzyme list

Gene	Protein
ACACA	acetyl-CoA carboxylase alpha
ACADVL	acyl-CoA dehydrogenase very long chain
ACLY	ATP citrate lyase
ACSL1 /2/3/4/5	acyl-CoA synthetase long chain family member 1/2/3/4/5
ALDH1L1 /2	aldehyde dehydrogenase 1 family member L1 /2
ALDOA /B/C	aldolase, fructose-bisphosphate A /B/C
BCAT1 /2	branched chain amino acid transaminase 1 /2
BCKDHA /B	branched chain keto acid dehydrogenase E1 subunit alpha /beta
BCKDK	branched chain keto acid dehydrogenase kinase
CS	citrate synthase
DGAT1/2	diacylglycerol O-acyltransferase 1 /2
FASN	fatty acid synthase
FBP1/2	fructose bisphosphate phosphatase
G6PC1	glucose-6-phosphatase catalytic subunit 1
GCK	glucokinase
GCKR	glucokinase regulator
GCSH	Glycine dehydrogenase (decarboxylating), mitochondrial
GLS1	glutaminase
GLS2	glutaminase 2
GLDC	Glycine cleavage system H protein, mitochondria
GLUD1 /2	glutamate dehydrogenase 1 /2
GPAM	glycerol-3-phosphate acyltransferase
GPT	glutamic--pyruvic transaminase
GPT2	glutamic--pyruvic transaminase 2
GYS1	glycogen synthase 1
H6PD	hexose-6-phosphate dehydrogenase/glucose 1-dehydrogenase
HSD17B8	hydroxysteroid 17-beta dehydrogenase 8
IDH1 /2	isocitrate dehydrogenase (NADP(+)) 1 /2
IDH3A	isocitrate dehydrogenase (NAD(+)) 3 catalytic subunit alpha
IDH3B	isocitrate dehydrogenase (NAD(+)) 3 non-catalytic subunit beta
IDH3G	isocitrate dehydrogenase (NAD(+)) 3 non-catalytic subunit gamma
LDHA /B/C	lactate dehydrogenase A /B/C
LDHAL6A /B	lactate dehydrogenase A like 6A /B
MDH1	malate dehydrogenase 1
ME1	malic enzyme 1
PDH	pyruvate dehydrogenase complex
PC	pyruvate carboxylase
PCK1 /2	phosphoenolpyruvate carboxykinase 1
PFKFB1 /2/3/4	6-phosphofructo-2-kinase/fructose-2,6-biphosphatase 1 /2/3/4
PFKM/L/P	phosphofructokinase
PGD	phosphogluconate dehydrogenase
PGLS	6-phosphogluconolactonase
PKLR	pyruvate kinase L/R
PKM 1/2	pyruvate kinase M 1/2
PPM1K	protein phosphatase, Mg2+/Mn2+ dependent 1K
PPP1R1A	protein phosphatase 1 regulatory inhibitor subunit 1A
SDHC	succinate dehydrogenase complex subunit C
SHMT2	serine hydroxymethyltransferase
TECR	trans-2,3-enoyl-CoA reductase
TKT	transketolase
TKTL1 /2	transketolase like 1 /2
